# The Effects of Hemisphere Dome Orientation on the Structure of Diamond-like Carbon Film Prepared Using Ion Beam Assisted Deposition

**DOI:** 10.3390/ma16051773

**Published:** 2023-02-21

**Authors:** Peng Shang, Yuanfei Ma, Zhenyun Zhang, Peng Sun, Huasong Liu, Hongchun Shi, Quan Lin, Tao Xue, Yiqin Ji

**Affiliations:** 1 GRINM Guojing Advanced Materials Co., Ltd., General Research Institute for Nonferrous Metals, Beijing 100088, China; 2 Tianjin Key Laboratory of Optical Thin Film, Tianjin Jinhang Technical Physics Institute, Tianjin 300308, China; 3 Center for Analysis and Tests, Tianjin University, Tianjin 300072, China

**Keywords:** hemisphere dome, diamond-like carbon, DLC film, ion beam assisted deposition, surface orientation

## Abstract

Diamond-like carbon (DLC) has attracted significant attention in the recent decades because of its unique properties and applications. Ion beam assisted deposition (IBAD) has been widely established in industry due to the advantages of easy handling and scalability. In this work, a hemisphere dome model is specially designed as a substrate. The influence of the surface orientation on the coating thickness, Raman I_D_/I_G_ ratio, surface roughness and the stress of the DLC films are examined. The reduction in the stress in the DLC films reflects the lower energy-dependence in diamond due to the varied sp3/sp2 fraction and columnar growth pattern. The variation of the surface orientation provides an efficient means of tailoring the properties and microstructure of the DLC films.

## 1. Introduction

Recently, diamond-like carbon (DLC) thin films have attracted significant attention because of their unique characteristics such as low friction, chemical inertness, high mechanical hardness, biocompatibility and wear resistance [1,2,3,4,5,6]. It can be used as the antireflective and protective film on optical windows [5,6,7,8,9,10]. Various techniques have been developed to produce DLC coatings such as physical vapor deposition, chemical vapor deposition, and their combination [1,2,3,4,5,6,7,8,9]. Among physical vapor deposition, Ion beam assisted deposition (IBAD) is one of the most widely established techniques for depositing a-C:H films in the industrial process [11,12,13,14,15] and possesses advantages such as low substrate temperature, high reliability and reproducibility [11,12,13,14,15,16]. Furthermore, with the rapid development of the high-speed flight platform, an urgent demand for the hard protective film on the surface of the optical dome has been proposed [5,6,17,18]. DLC coating is competent in achieving excellent optic properties and environmental stability due to its uniform performance over the dome surface [19]. Researchers revealed that the composition, structure and mechanical properties of the as-deposited DLC coatings could be seriously affected by many growth parameters in the deposition process, and the orientation of substrates is a key factor [20].

Previous investigation [21,22,23,24] suggested how coating thickness, microhardness and bonding ratios were affected by the surface’s shielding using a specially designed double V-shaped jig, L-shaped jig, and O-shaped jig. For example, Ding et al. [22] deposited the amorphous carbon films using the unbalanced magnetron sputtering method and determined the influence of substrate geometric parameter and geometric aspect ratio on the coating characteristics using a specially designed double V-shaped jig. The investigation revealed that the geometry of a substrate could vary the ion impingement angle and ion energy during deposition on the substrate surfaces, which results in a change in the structure and characteristics of film. Nelson et al. [23] demonstrated that the carbon thin films deposited through Plasma-Enhanced Chemical Vapor Deposition (PECVD) on horizontally placed substrates exhibit apparently different properties, such as surface roughness, thickness and chemical bonding from those deposited carbon thin films to vertically placed substrates. Jonathan Laumerl [24] assessed the role of the geometry associated with the underlying substrate in shaping the thin films of carbon deposited onto curved surfaces, i.e., rings, and flat substrates using magnetron sputtering and plasma-enhanced chemical vapor deposition techniques.

In this paper, the influences of the characteristics of the nanostructured DLC coating on the hemisphere dome orientation is directly investigated. A deeper understanding of these local variations will benefit the optimization of coatings for complex substrate geometries. In this case, a hemisphere dome model is specially designed and employed. The DLC films are deposited using IBAD and the influence of the dome surface orientation on the coating thickness, Raman I_D_/I_G_ ratio, surface roughness and the micro-structure are presented in this work.

## 2. Materials and Methods

**Figure 1:**
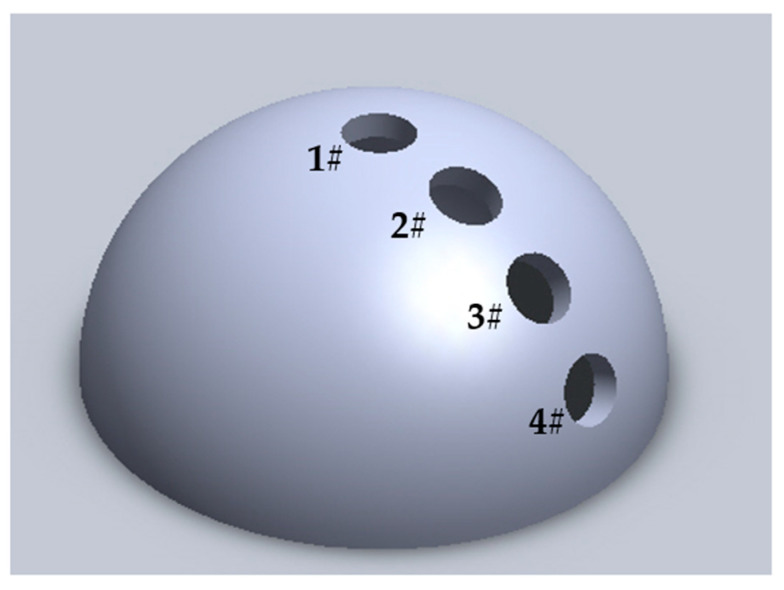
Schematic diagram of the designed 3D hemisphere dome model (1#-sample 1, 2#-sample 2, 3#-sample 3, 4#-sample 4).

**Figure 2:**
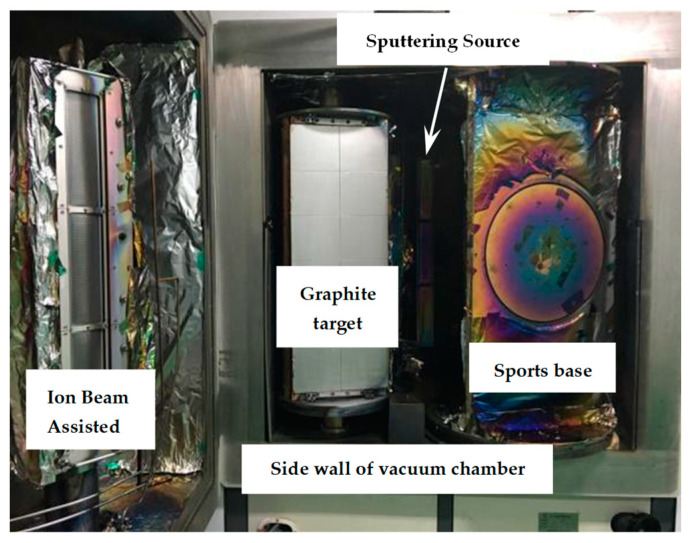
Internal structure diagram of ion source sputtering coating equipment.

**Figure 3:**
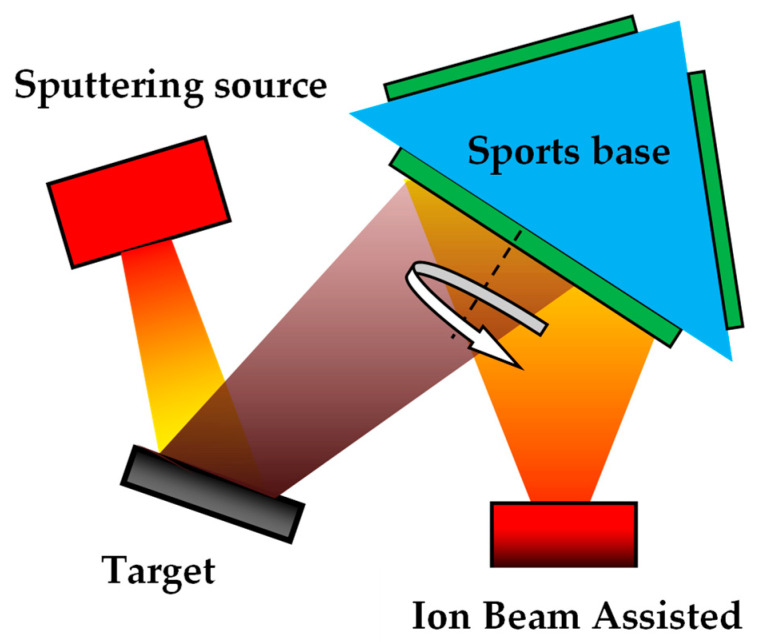
Schematic diagram of DLC film grown by ion beam sputtering method.

The hemispherical dome model with a diameter of 240 mm was machined from aluminum for the subsequent DLC films deposition, as shown in Figure 1. Four opened holes distribute evenly on the surface for placing the test pieces. All samples were ultrasonically cleaned in acetone for 10 min before being placed in the holes of spherical dome model. Then, the DLC films were deposited on the polished Si and BK7 pieces using IBAD. Internal structure diagram of ion beam sputtering coating equipment and schematic diagram of DLC film grown using ion beam sputtering method were shown in Figure 2 and Figure 3, respectively.

The graphite target with 99.5% purity was placed in the deposition chamber, and Ar (99.999%) as the discharge gas. During the deposition of DLC film, CH_4_ (99.99%) was used as a reactive gas with a flow of 40 sccm. The vacuum system was formed using a turbo-molecular pump and an auxiliary mechanical pump. The chamber was evacuated to a base vacuum of approximately 6 × 10^−6^ Torr, and a common operating vacuum level was approximately 2 × 10^−4^ Torr. All samples were prepared using the optimized process in our experiments. The DLC films were obtained under typical experimental parameters: beam current I_beam_ = 400 mA, beam voltage V_beam_ = 600 V, RF power P_RF_ = 250 W, Ar gas flow rate 70 sccm. Prior to deposition, all substrates were sputter cleaned in pure Ar plasma at beam voltage 1000 V, beam current 300 mA for 5 min. The temperature on the surface of the film during deposition was 60 ± 10 °C in situ monitored using thermocouple measurements. The IBAD equipment was manufactured by Chengdu Xinghangfan Vacuum Technology Co., Ltd. (Chengdu, China).

The physical thickness was controlled using deposition time based on the stable deposition rate. It was about 1.5 nm/min. The cross-section of the film was studied using scanning electron microscopy (SEM), using a Hitachi S-4800 electron microscope (Tokyo, Japan). The testing sample was mounted on the sample stud by means of double-sided adhesive tape. The SEM measurements were performed at an accelerating voltage of 5 kV. A thin layer of gold was sputtered prior to the SEM measurement. The optical constants of films were calculated from infrared transmittance spectra in WVASE32 software (J. A. Woollam. Co., Lincoln, NE, USA). A white light interferometer (ContourGT-K, Germany BRUKER company, Karlsruhe, Germany) of which the vertical resolution can reach 0.01 nm was used to obtain the surface morphology of all DLC samples.

The key property of interest in the DLC films is the relative amount of sp^3^/sp^2^ bonded carbon present in the films. The observed D and G peaks of varying integral intensity, position and width dominating various carbon coatings having different sp^3^/sp^2^ ratios continues to intrigue scientists to this day. Raman spectroscopy is still widely used to derive meaningful information on the sp^3^/sp^2^ content in the DLC coatings. All the spectra indicated the presence of two broad peaks located at ~1360 cm**^−^**^1^ (D-line) and ~1550 cm**^−^**^1^ (G-line). In this paper, Raman spectra were acquired at 532 nm wavelength with a Nd:YAG laser using Renishaw inVia equipment. The system set up with 2400 grooves/mm grating and 50× objective lens. The spectral resolution was about 1 cm**^−^**^1^. The laser power was kept at 1% (0.63 mW). The Raman spectra in the 1000 cm**^−^**^1^~2000 cm**^−^**^1^ region was acquired in order to investigate the internal bonding in the DLC films. In order to make the experimental results more accurate, we tested three different positions on the surface of each sample. The two Gaussian curves were fitted using commercially available software (OriginPro 8.5.1, OriginLab® corporation, Northampton, MA, USA) to identify peak positions and intensities. Then the average value was obtained. The residual stress f σ of the DLC thin films was evaluated by measuring the change in their curvature, according to Stoney’s expression [25]:

(1) $$\sigma =E_{s}t_{s}^{2}/6\left(1-\vartheta_{s}\right)t_{f},$$

where E_s_ and υ_s_ were the Young modulus and the Poisson ratio, respectively; t_s_ and t_f_ were the thickness of the substrate and the film, respectively; R was the spherical radius of the curvature of the disk-shaped glass substrate. If r was the radius of the coated area and Δδ was the vertical displacement, then the curvature radius is approximated using R = r^2^/2Δδ. The deposited film thickness t_f_ was determined using SEM. A phase-shifting Twyman-Green interferometer was used to determine the vertical displacement Δδ by measuring changes in the fringes caused by the deposited thin film. Specifically, the uncoated or coated substrate is mounted vertically at the centre of the sample holder used as the test plate. The sample holder has a circular aperture through which the incident beam passes and is then reflected onto the substrate surface. After two beam reflections, interference fringes of equal thickness arise between the surface of the reference plate and the surface of the substrate. A computer-controlled image frame grabber captures the active images and digitizes them. The tensile stress was herein taken as positive and the compressive stress as negative. The substrate used to measure stress was BK7 (r: 25.4 mm, t_s_: 1.5 mm, E_s_: 81 GPa, υ_s_: 0.208). All measurements were conducted in air at room temperature.

## 3. Results and Discussion

### 3.1. Thickness Measurements with SEM

**Figure 4:**
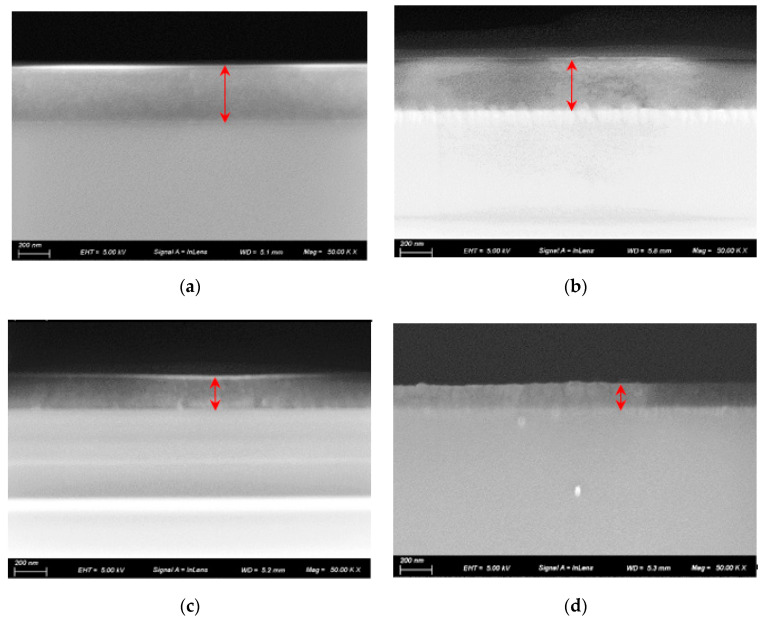
SEM images of the cross section of samples. (**a**) sample 1; (**b**) sample 2; (**c**) sample 3 (**d**) sample 4.

**Figure 5:**
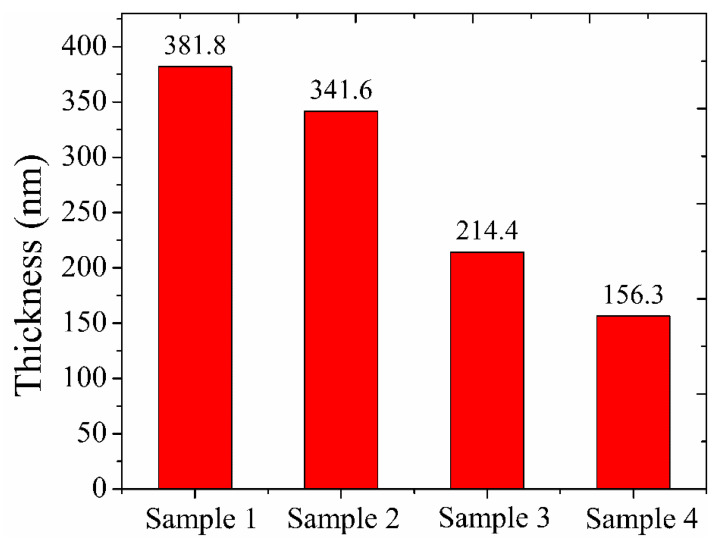
Coating thickness of samples derived from SEM images.

The variation of the cross-section of samples is presented in Figure 4. SEM analysis of the sectioned samples showed that the edge of the dome (Figure 4d) had a columnar growth pattern throughout the silicon and carbon layers, compared to the top sample (Figure 4a) which had a much more uniform coating with compact microstructure and clear transition lines between layers. Coating thickness of samples derived from SEM images are shown in Figure 5. It can be found that the thickness of the DLC film layer had a large change in comparison with the change in the position of the dome model. At the top of the dome model, the coating thickness was ~381nm and then drastically decreased to ~156 nm at the edge.

### 3.2. Studies of Surface Characterization

**Figure 6:**
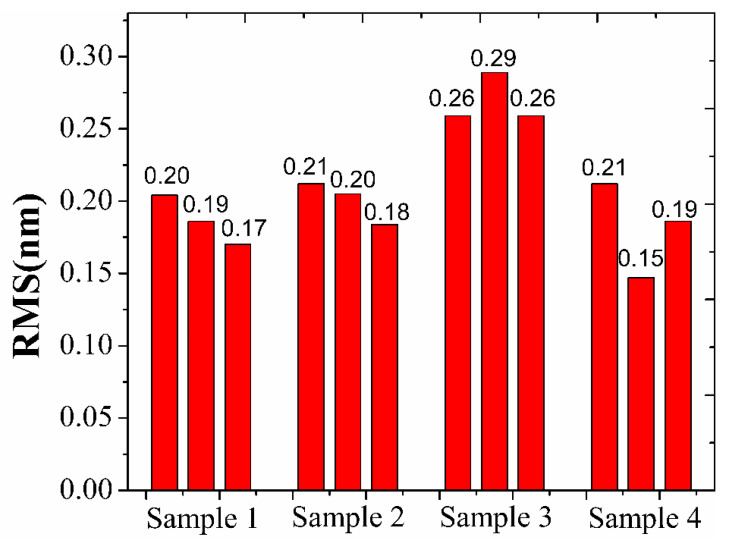
The surface roughness of the DLC films at different model positions.

Figure 6 presents the surface roughness of the DLC films at different model positions. The root mean square (RMS)of the surface roughness was measured in three different points in each sample and the average RMS roughness was analyzed. Surface topography test results show that the surface roughness, Ra, depends strongly on its location on the dome model. The average surface roughness of sample 1, sample 2, sample 3 and sample 4 are 0.19 nm, 0.20 nm, 0.27 nm and 0.18 nm, respectively. The roughnesss gradually increases from sample 1 to sample 3 and reaches the maximum value (~0.27 nm) at the location of sample 3. Finally, the roughnesss decreases monotonically from sample 3 to sample 4. This roughness evolution of film can be attributed to the combination of the hydrogen etching and the surface diffusion during the process. Specifically, the hydrogen etching and sputtering increases with the change in inspected position from the top to edge of the dome during the film growth, which leads to an increase in surface roughening. With the increasing of the deposition angles, the surface diffusion effect enhances, which leads to the decrease in the surface roughness of sample 4.

### 3.3. Raman Studies of Diamonds at Various Temperatures

**Figure 7:**
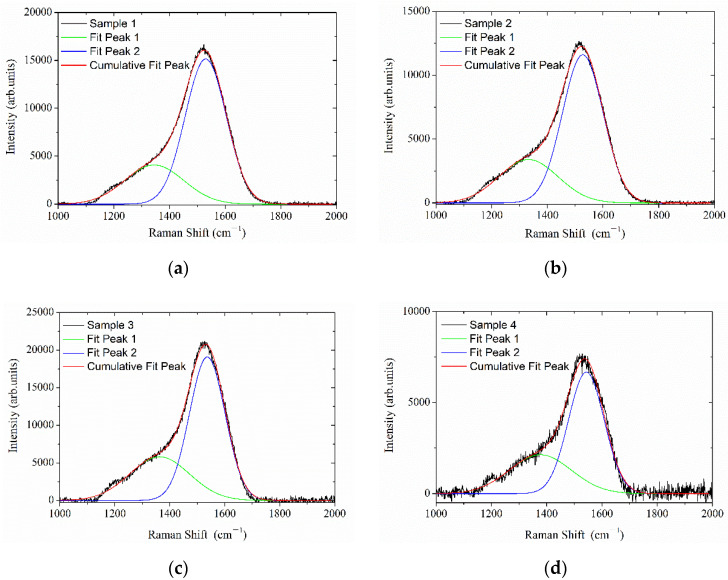
Raman spectrum of DLC films collected at excitation wavelength of 532 nm. (**a**) sample 1; (**b**) sample 2; (**c**) sample 3 (**d**) sample 4.

Raman backscattering spectroscopy is a fast and non-destructive method used to study the characteristics of amorphous carbon. The Raman spectra in the 1000 cm^−1^~2000 cm^−1^ region were acquired in order to investigate the internal bonding in the DLC films. The Raman spectra are presented in Figure 7. The G band at approximately 1560 cm^−1^ is associated with the sp^2^-bonded carbon, while the D band appearing at approximately 1360 cm^−1^ is attributed to the disorder allowed at the zone edge mode of graphite. The ratio of the integral intensities of the disorder D peak to the graphite G peak I_D_/I_G_ is used to describe the degree of derangement in sp^2^-bonded carbon materials [26]. The relatively high value of I_D_/I_G_ could be attributed to the sp^2^-bonding-dominated structure of the as-synthesized film.

The influence of substrate surface orientation on the I_D_/I_G_ ratio is indicated in Table 1. The average I_D_/I_G_ ratios of sample 1, sample 2, sample 3 and sample 4 are 0.37, 0.41, 0.52 and 0.56, respectively. Only a slight decrease was observed between sample 2 and sample 1, whilst a dramatic reduction for sample 4 which corresponds to a surface orientation angle of approximately 67.5° was observed. It indicated that sample 4 had a highest average sp^2^ content compared to sample 1, sample 2 and sample 3. The difference can be explained through the sub-plantation model during film growth. Specifically, as the ions travel normally to the substrate surface, the majority of the kinetic energy is employed to penetrate the surface and the surface diffusion may decrease. As the direction of the impinging ion deviates from the normal line, more total energy will be required for the ion to reach the same depth. It is expected that the density of the structure decreased at a more oblique angle, which results in the poor coating quality. Therefore, during the deposition of a-C:H layers, the ions have less kinetic energy to penetrate the surface in order to arrive at the location of sample 4 than that of sample 1, which results in the increasing of I_D_/I_G_ ratio.

### 3.4. Residual Stress

Table 2 shows the results of the measured residual stress. It can be found that the stress of the deposited films varied with surface orientation. They were compressive regardless of film thickness. The maximum residual stress is about 875 MPa and occurs at the top of the dome model. The compressive stress decreases from the top to the edge. Such variation is closely related to the differences in the texture and sp**^3^**/sp**^2^** bonding ratios of the coatings. As shown in Figure 2, sample 4 was placed on the edge of the window. It had a columnar growth pattern, compared with the top of sample 1, which had a much more uniform coating with compact microstructure. The residual stress was then released. Furthermore, the reduced sp^3^ content in sample 4, which can be linked to a softer film, leads to a lowered stress value compared to that of sample 1 and sample 2.

### 3.5. Optical Constant

**Figure 8:**
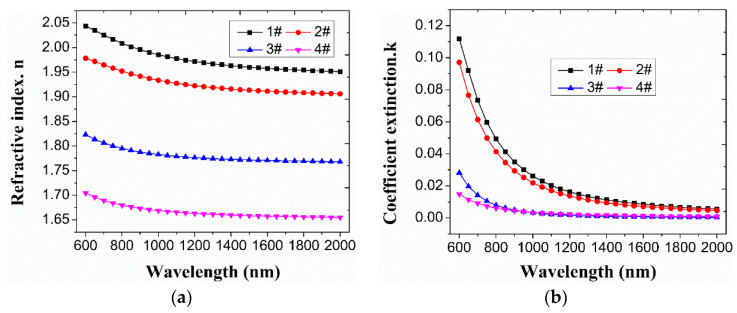
The variation of optical constants in the 600~2000 nm band. (**a**) Refractive index; (**b**) Coefficient extinction.

The variation of optical constants in the 600~2000 nm band is shown in Figure 8. It can be seen that the refractive index of the film decreases gradually as the position changes in the sample from the top to the bottom of the dome model. The refractive index ranges from ~2.05@2000 nm to ~1.66@2000 nm. The change in the extinction coefficient is relatively complex. The extinction coefficients of sample 1 and sample 2 are in the same order of magnitude. Compared to sample 1 and sample 2, sample 3 and sample 4 have a significant reduction, which may be closely related to the increase in hydrogen content in DLC film. Specifically, CH_4_ is very efficiently decomposed through a sequence of reactions and collisions in which H appears as the most abundant product at the process end. The increase in hydrogen content is beneficial to the decrease in the extinction coefficient of DLC films.

## 4. Conclusions

In summary, the variations in the coating characteristics relating to the substrate surface orientation is studied. The effects of the substrate orientation on the DLC film structure were confirmed. The SEM showed that the samples display a significant change of up to 50% in microstructure and thickness differences. The film presented compressive stress with a decreased trend from the top to the bottom. The roughness, RMS, varied from 0.18 nm to 0.27 nm between samples shown using the optical profilometer. Raman spectroscopy highlighted variations in the sp^3^/sp^2^ bonding ratios. The variation of the surface orientation provides an efficient means of tailoring the properties and microstructure of the DLC films. In the future, the swing clamp system is expected to improve the performance of the DLC film.

## Figures and Tables

**Table 1:** Average deconvolution Raman data of DLC coatings deposited on different deposited samples.

Samples	Peak Type	Peak Position (cm^−1^)	FWHM (cm^−1^)	I_D_/I_G_
1	D	1347	246	0.37
	G	1530	174	
2	D	1336	251	0.41
	G	1528	172	
3	D	1360	258	0.52
	G	1536	155	
4	D	1371	267	0.56
	G	1543	152	

**Table 2:** Residual stress in film evaluated by measuring the curvature change using Stoney’s equation.

Sample	Film Thickness/nm	Vertical Displacement/nm	Stress/Mpa
Sample 1	381.8	−1155.30	−875
Sample 2	341.6	−704.05	−596
Sample 3	214.4	−131.46	−177
Sample 4	156.3	−42.34	−78

## Data Availability

Not applicable.

## References

[1] Grill A. (1999). Diamond-like carbon: State of the art. Diam. Relat. Mater..

[2] Robertson J. (2002). Diamond-like amorphous carbon. Mater. Sci. Eng. R Rep..

[3] Lifshitz Y. (1999). Diamond-like carbon—Present status. Diam. Relat. Mater..

[4] Erdemir A., Donnet C. (2006). Tribology of diamond-like carbon films: Recent progress and future prospects. J. Phys. D Appl. Phys..

[5] Xu D., Qing L., Xiang K. (2009). Optical and electrical properties evolution of diamond-like carbon thin films with deposition temperature. Chin. Phys. Lett..

[6] Hainsworth S.V., Uhure N.J. (2007). Diamond like carbon coatings for tribology: Production techniques, characterisation methods and applications. Int. Mater. Rev..

[7] Xu J., Fan H., Liu W., Hang L. (2008). Large-area uniform hydrogen-free diamond-like carbon films prepared by unbalanced magnetron sputtering for infrared anti-reflection coatings. Diam. Relat. Mater..

[8] Song J.S., Park Y.S., Kim N.H. (2021). Hydrophobic anti-reflective coating of plasma-enhanced chemical vapor deposited diamond-like carbon thin films with various thicknesses for dye-sensitized solar cells. Appl. Sci..

[9] Swec D.M., Mirtich M.J. (1989). Diamondlike carbon protective coatings for optical windows. Window Dome Technol. Mater..

[10] Harshavardhan K.S., Iyer S.B. (1994). Diamond-like carbon coating for optical windows. IETE J. Res..

[11] Chowdhury S., Laugier M.T., Rahman I.Z. (2004). Characterization of DLC coatings deposited by rf magnetron sputtering. J. Mater. Process. Technol..

[12] Sanchez N.A., Rincon C., Zambrano G., Galindo H., Prieto P. (2000). Characterization of diamond-like carbon (DLC) thin films prepared by rf magnetron sputtering. Thin Solid Film..

[13] Li D.J., Cui F.Z., Gu H.Q. (1999). Studies of diamond-like carbon films coated on PMMA by ion beam assisted deposition. Appl. Surf. Sci..

[14] Kitagawa T., Yamada I., Toyoda N., Tsubakino H., Matsuo J., Takaoka G.H., Kirkpatrick A. (2003). Hard DLC film formation by gas cluster ion beam assisted deposition. Nucl. Instrum. Methods Phys. Res. Sect. B Beam Interact. Mater. At..

[15] Li D.J., Cui F.Z., Gu H.Q. (1999). Diamond-like carbon coating on poly (methylmethacrylate) prepared by ion beam deposition and ion beam-assisted deposition and its effect on cell adhesion. J. Adhes. Sci. Technol..

[16] Kimock F.M., Knapp B.J. (1993). Commercial applications of ion beam deposited diamond-like carbon (DLC) coatings. Surf. Coat. Technol..

[17] Mangla O., Roy S. (2021). Synthesis of nano-diamond-like carbon for protective optical window coating applications. Bull. Mater. Sci..

[18] Sun P., Liu H., Cheng J., Liu D., Leng J., Ji Y. (2022). Germanium carbon (Ge1-xCx)/diamond-like carbon (DLC) antireflective and protective coating on zinc sulfide window. Opt. Mater..

[19] Li Z.L., Fu X.H., Lu J., Yang Y.L., Sun D.G. (2013). Modelling and Optimization of DLC Film Thickness Variation for PECVD Processes. Key Eng. Mater..

[20] Cuomo J.J., Pappas D.L., Lossy R., Doyle J.P., Bruley J., Di Bello G.W., Krakow W. (1992). Energetic carbon deposition at oblique angles. J. Vac. Sci. Technol. A Vac. Surf. Film..

[21] Bobzin K., Bagcivan N., Goebbels N., Yilmaz K. (2009). Effect of the substrate geometry on plasma synthesis of DLC coatings. Plasma Process. Polym..

[22] Ding X.Z., Zeng X.T., Hu Z.Q. (2004). Substrate geometry effect on the uniformity of amorphous carbon films deposited by unbalanced magnetron sputtering. Thin Solid Film..

[23] Nelson N., Rakowski R.T., Franks J., Woolliams P., Weaver P., Jones B.J. (2014). The effect of substrate geometry and surface orientation on the film structure of DLC deposited using PECVD. Surf. Coat. Technol..

[24] Laumer J., O’Leary S.K. (2019). An adhesion analysis of thin carbon films deposited onto curved and flat Ti6Al4V substrates using rf magnetron sputtering and plasma enhanced chemical vapor deposition techniques. J. Mater. Sci. Mater. Electron..

[25] Stoney G.G. (1909). The tension of metallic films deposited by electrolysis. Proc. R. Soc. London. Ser. A Contain. Pap. A Math. Phys. Character.

[26] Annett D., Andy E., Stefan S., Christian S., Steffen W. (2020). Laser structuring of hydrogenated DLC scaffolds: Raman spectroscopy and nanotribology. Diam. Relat. Mater..

